# Preoperative levels of folate receptor-positive circulating tumor cells in different subtypes of early-stage lung adenocarcinoma: Predictive value for determining extent of surgical resection

**DOI:** 10.3389/fonc.2023.1119807

**Published:** 2023-04-17

**Authors:** Chao Zhou, Ran Zhao, Ruiying Zhao, Ansheng Wang, Wentao Li

**Affiliations:** ^1^ Department of Thoracic Surgery, Shanghai Chest Hospital, School of Medicine, Shanghai Jiao Tong University, Shanghai, China; ^2^ Department of Thoracic Surgery, ShuYang Hospital of Traditional Chinese Medicine, Suqian, China; ^3^ Department of Pathology, Shanghai Chest Hospital, School of Medicine, Shanghai Jiao Tong University, Shanghai, China; ^4^ Department of Thoracic Surgery, The First Affiliated Hospital of Bengbu Medical College, Bengbu, China

**Keywords:** pathological subtype, intraoperative frozen sections, diagnosis, sensitivity, specificity, lung adenocarcinoma

## Abstract

**Background:**

The objective was to measure the correlations of preoperative levels of folate receptor-positive circulating tumor cells (FR^+^CTCs) with clinical characteristics and histologic subtype in early-stage lung adenocarcinoma, and to determine the predictive value of FR^+^CTC level in preoperative determination of the extent of surgical resection.

**Patients and methods:**

In this retrospective, single-institution, observational study, preoperative FR^+^CTC levels were measured *via* ligand-targeted enzyme-linked polymerization in patients with early-stage lung adenocarcinoma. Receiver operating characteristic (ROC) analysis was used to identify the optimal cutoff value of FR^+^CTC level for prediction of various clinical characteristics and histologic subtypes.

**Results:**

No significant difference in FR^+^CTC level was observed among patients with adenocarcinoma *in situ* (AIS), minimally invasive adenocarcinoma (MIA), and invasive adenocarcinoma (IAC) (*P* = 0.813). Within the non-mucinous adenocarcinoma group, no difference was observed among patients with tumors whose predominant growth patterns were lepidic, acinar, papillary, micropapillary, solid, and complex gland (*P* = 0.053). However, significant differences in FR^+^CTC level were observed between patients with and without the micropapillary subtype [11.21 (8.22-13.61) *vs*. 9.85 (7.43-12.63), *P* = 0.017], between those with and without the solid subtype [12.16 (8.27-14.90) *vs*. 9.87 (7.50-12.49), *P* = 0.022], and between those with any of the advanced subtypes (micropapillary, solid, or complex glands) vs. none of these [10.48 (7.83-13.67) *vs*. 9.76 (7.42-12.42), *P* = 0.032]. FR^+^CTC level was also correlated with degree of differentiation of lung adenocarcinoma (*P* = 0.033), presence of visceral pleural invasion (VPI) of lung carcinoma (*P* = 0.003), and lymph node metastasis of lung carcinoma (*P* = 0.035).

**Conclusion:**

FR^+^CTC level is of potential predictive value in determining the presence of aggressive histologic patterns (micropapillary, solid, and advanced subtypes), degree of differentiation, and occurrence of VPI and lymph node metastasis in IAC. Measurement of FR^+^CTC level combined with intraoperative frozen sections may represent a more effective method of guiding resection strategy in cases of cT1N0M0 IAC with high-risk factors.

## 1 Introduction

Lung cancer is the second-most common cancer worldwide and the leading cause of cancer morbidity and mortality in men (1). Non-small cell lung cancer (NSCLC) accounts for approximately 85% of all lung cancers, and adenocarcinoma is the most common histologic type of NSCLC (2). Lung adenocarcinoma can be further subdivided into preinvasive lesions [atypical adenomatous hyperplasia (AAH) and adenocarcinoma *in situ* (AIS)], minimally invasive adenocarcinoma (MIA), and invasive adenocarcinoma (IAC), according to the degree of invasion (3). The different subtypes of lung adenocarcinoma strongly impact the choice of surgical methods and the prognosis of the patient. Previous studies have reported that in stage I lung adenocarcinoma, patients with the AIS and MIA subtypes have a 5-year disease-free survival (DFS) rate close to 100%, while this rate is only 38%–86% for those with IAC (4, 5).

Lobectomy remains the standard surgical treatment for lung adenocarcinoma. However, non-IAC patients also have the option of limited surgical resection (6). Several prospective, multicenter clinical studies have investigated the effect of lobectomy vs. sublobectomy on the survival rates of AIS and MIA patients. They have found that sublobectomy is the optimal choice, producing similar survival rates along with better quality of life (7, 8). If we could precisely determine the degree of invasiveness of lung adenocarcinoma intraoperatively, the less damaging sublobectomy would be feasible in more cases, based on guaranteed curative resection. However, Yeh et al. have reported that interobserver agreement in terms of discriminating between AIS, MIA, and invasive adenocarcinomas using frozen sections (FSs) is not satisfactory (*κ* = 0.378, fair agreement) (9). As such, the identification of AIS and MIA by examination of intraoperative FSs is not recommended by the prevailing guidelines.

Non-mucinous invasive lung adenocarcinoma is mainly divided into lepidic, acinar, papillary, micropapillary, and solid predominant patterns; this pattern is increasingly considered to be a powerful predictor of prognosis in lung adenocarcinoma (10). IAC, with a predominant lepidic pattern, has been found to reflect indolent behavior in terms of the biological behavior of the tumor, which is associated with a favorable prognosis (11). In contrast, studies have shown that prognosis is significantly worse in the case of sublobectomy than in the case of lobectomy among IAC patients with micropapillary composition ≥5% (11–13). Furthermore, the presence of micropapillary or solid components has been found to be an independent predictor for unfavorable recurrence-free survival (RFS) rates in cT1N0M0 lung adenocarcinoma patients undergoing lobectomy (14). Preoperatively and intraoperatively, accurate histologic subtyping is helpful to enable thoracic surgeons to decide on the extent of surgical resection. The accuracy of FS for prediction of the predominant histologic subtype is not satisfactory (*κ* = 0.565, moderate agreement), and the sensitivity of FS analysis, in terms of the presence or absence of micropapillary and solid patterns, is poor (micropapillary: 37%, *κ* = 0.321, fair agreement; solid: 69%, *κ* = 0.67, substantial agreement) (9).

To date, studies on intraoperative FS have demonstrated low consistency in the determination of pathological subtype (9, 15–17). Therefore, it is desirable to identify preoperative biomarkers that can distinguish the histologic patterns of early-stage lung adenocarcinoma.

Circulating tumor cell (CTC) level has proven utility in the early diagnosis of cancer, in monitoring recurrence and metastasis, in determining the prognosis of surgical and systemic interventions, and in the selection of postoperative adjuvant therapy (18). Folate receptor-positive CTCs (FR^+^CTCs) are a type of circulating rare cell with high expression of FRs on the cell surface; levels of these cells can be reliably quantified using commercially available kits (19). Lung cancer patients of all stages have been found to exhibit significantly higher FR^+^CTC levels than patients with benign lung disease and healthy subjects. Studies have also shown that FR^+^CTC level can be used to diagnose lung cancer with high sensitivity (79.6%) and specificity (88.2%) (19–21). Furthermore, FR^+^CTC level is significantly higher in patients with MIA and IAC than in AIS patients (22). However, the correlation between FR^+^CTCs and the histologic patterns of cTis-T1N0M0 lung adenocarcinoma remains to be explored, and the question of whether FR^+^CTC level can help thoracic surgeons to determine the extent of surgical resection required for early-stage lung adenocarcinoma is worthy of investigation.

## 2 Patients and methods

### 2.1 Study design

This retrospective, single-institution study was conducted at Shanghai Chest Hospital from April 2021 to August 2022. A total of 1,835 patients with suspected lung cancer who were recommended for radical surgery were included in the study. The inclusion criteria were as follows: 1) age between 18 and 80 years old; 2) clinical stage Tis-T1N0M0 lung adenocarcinoma according to the eighth edition of the Tumor Node Metastasis (TNM) Classification for Lung Cancer (23); and 3) FR^+^CTC test conducted prior to the operation. The exclusion criteria were as follows: 1) multiple primary lung cancer (MPLC); 2) a history of lung cancer or other malignancies; 3) prior antitumor therapy (including neoadjuvant therapy); 4) non-lung adenocarcinoma; and 5) incomplete clinical information.

For all patients, pathological evaluation of the diseased tissue was conducted by a professional pathologist according to the IASLC/ATS/ERS classification of lung adenocarcinoma (24). The Ethics Committee of the Shanghai Chest Hospital approved the study.

### 2.2 FR^+^CTC analysis

A sample of 3 ml of peripheral blood was collected from each participant using an ethylenediaminetetraacetic acid (EDTA) anticoagulant vacuum tube, stored at 4°C, and processed within 24 h. FR^+^CTC detection was performed using a Folate Receptor-positive Cell Detection Kit (GenoSaber, Shanghai, China). Two steps were followed to enrich CTCs: erythrocytes were first lysed, and leukocytes were subsequently eliminated with reference to the manufacturer’s specified protocol. The labeled FR^+^CTCs were enumerated *via* quantitative polymerase chain reaction (PCR) using the proprietary ligand-targeted PCR method (21). A series of standards containing oligonucleotides (10^−14^ to 10^−9^ to 2 to 2 × 10^5^ M, corresponding to FU/3 ml blood) were used for quantification of FR^+^CTCs. The number of folate-receptor units (FU) per 3 ml of peripheral blood was calculated from the standard curve and was used to determine the FR^+^CTC level in each sample.

### 2.3 Postoperative pathological examination

Pathological sections were fixed by embedding in paraffin and stained with hematoxylin–eosin (HE). All components with a proportion greater than 5% were recorded in 5% increments. The pathological images were independently interpreted by two pathologists with more than 5 years of experience. If there was any dispute, the diagnosis was made by reading the images together under a multihead microscope. Nodules were reclassified according to the 2021 WHO histological classification of lung tumors and divided into AIS, MIA, and IAC (**Supplementary Figures 1A–C**: A and C, ×200; B, ×50). Cases of IAC were further subdivided into five types according to the component with the highest proportion: lepidic, acinar, papillary, micropapillary, or solid predominant (**Supplementary Figures 1D–H**, ×200).

### 2.4 Data collection

Data were collected from clinical records using the standard case report form (CRF). Data including demographic characteristics, clinical symptoms, types of surgery undergone, genetic testing, and CT imaging information.

### 2.5 Statistical analysis

Descriptive statistics are expressed in the form of medians (interquartile range) for continuous variables and in the form of counts (percentages) for categorical variables. Continuous variables were compared using the Mann–Whitney test for comparisons between two groups or the Kruskal–Wallis test for comparisons among three groups. Categorical variables were compared using the chi-square test. Receiver operating characteristic (ROC) analysis was used to determine the optimal threshold of FR^+^CTC level, and the associated specificity and sensitivity, for classifying cases according to growth pattern, degree of differentiation, lymph node metastasis, and visceral pleural invasion (VPI). All statistical analyses were performed using STATA version 16 SE (Stata Corporation, TX, USA). All *P*-values were calculated based on two-sided testing. A *P*-value <0.05 was considered to represent statistical significance.

## 3 Results

### 3.1 Characteristics of the patients

**Figure 1:**
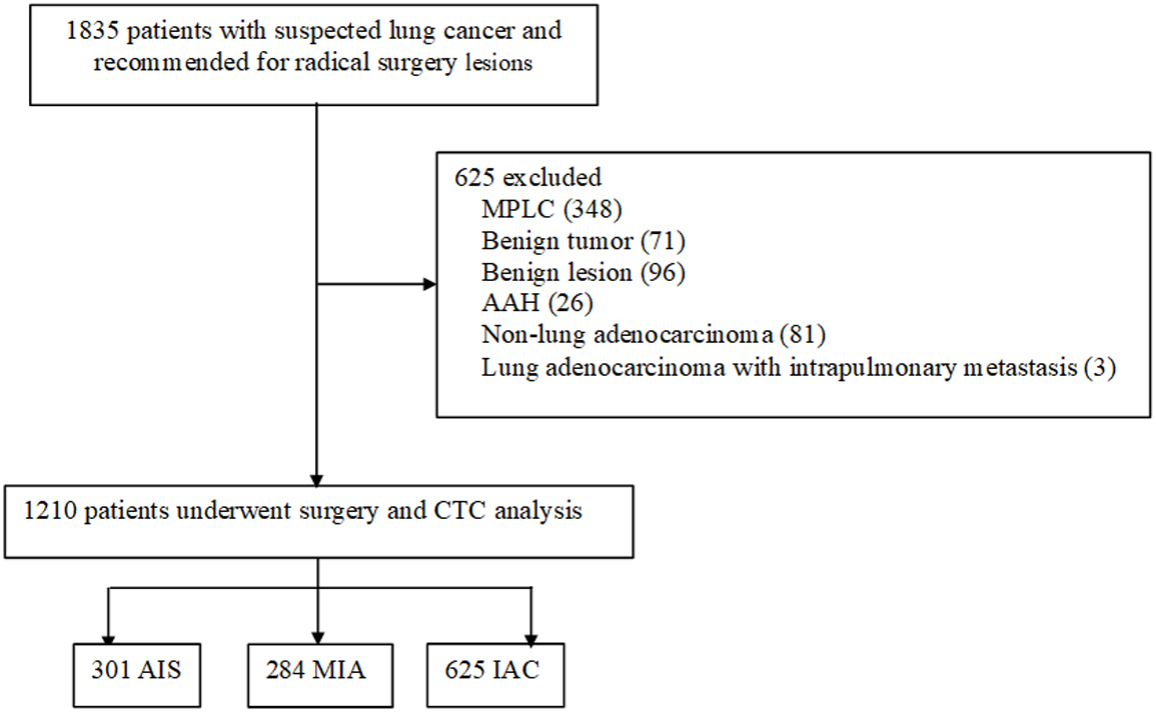
Flowchart of patient screening.

A total of 1,835 patients admitted for surgical operation during the study period were reviewed in this study. Of these, 625 patients were excluded: 348 had MPLC, 71 had a benign tumor, 96 had non-tumorous benign lesions, 26 had AAH, 81 had non-lung adenocarcinoma, and 3 had lung adenocarcinoma with intrapulmonary metastasis. Thus, a total of 1,210 patients were included in the final analysis (**Figure 1**). Among these patients, 438 were men (36.17%), and 519 (42.86%) were older than 60 years of age. Additionally, of the 1,210 patients included, 301 (24.9%) were AIS cases, 284 (23.5%) were MIA, and 625 (51.6%) were IAC. The demographic and clinical characteristics of the participants are summarized in **Supplementary Table 1**.

### 3.2 Correlation of FR^+^CTC level with pathological type

**Figure 2:**
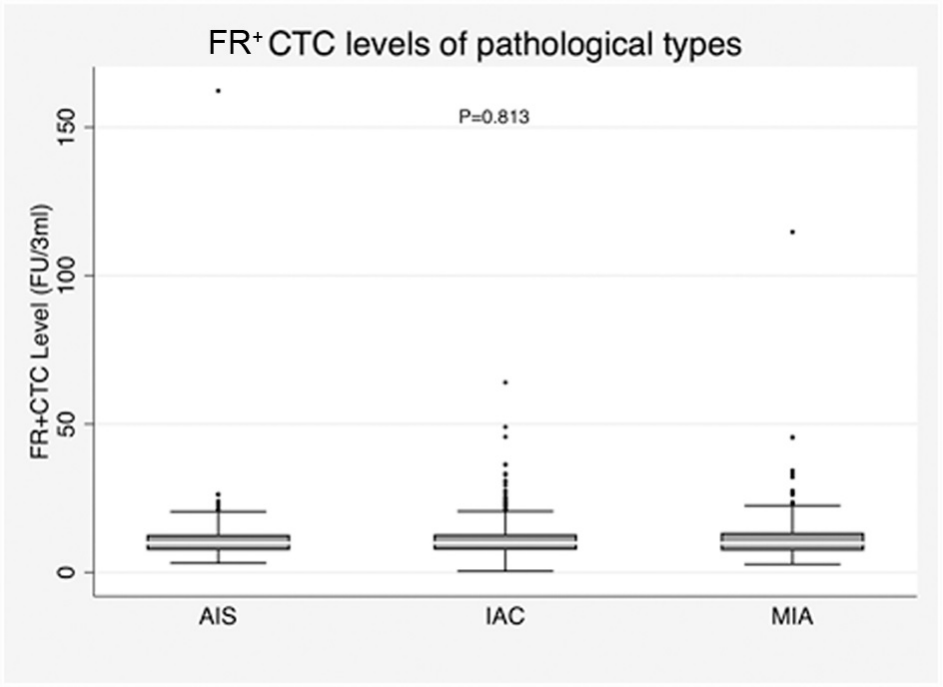
Correlation of FR^+^CTC level with pathological type across all included patients.

The median (IQR) FR^+^CTC level in the AIS, MIA, and IAC groups was 9.92 (7.45, 12.74) FU/3 ml, 9.86 (7.36, 13.4) FU/3 ml, and 9.93 (7.59, 12.9) FU/3 ml, respectively. No significant differences in FR^+^CTC level were observed among the AIS, MIA, and IAC groups (*P* = 0.813) (**Figure 2**; **Supplementary Table 1**).

### 3.3 Correlation of FR^+^CTC level with growth pattern subtype in IAC patients

**Figure 3:**
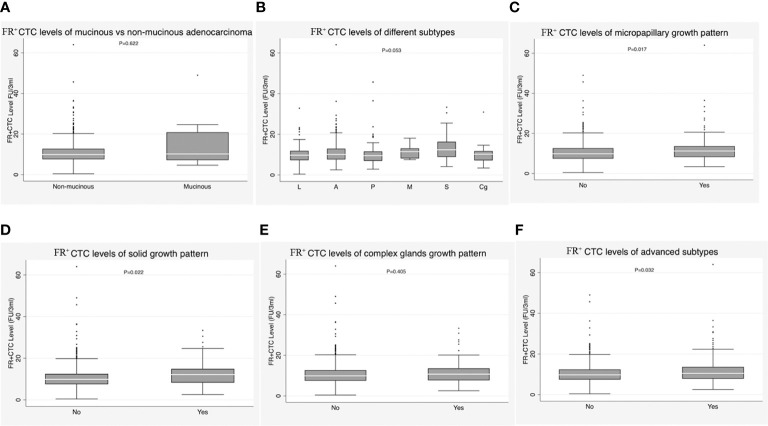
**(A)** Correlation of FR^+^CTC level with mucinous adenocarcinoma vs. non-mucinous adenocarcinoma in the invasive adenocarcinoma (IAC) group. **(B)** Correlation of FR^+^CTC level with lepidic subtype, acinar subtype, papillary subtype, micropapillary subtypes, solid subtype, and complex glands in the IAC group. **(C)** Correlation of FR^+^CTC level with presence vs. absence of micropapillary components in the IAC group. **(D)** Correlation of FR^+^CTC level with presence vs. absence of solid components in the IAC group. **(E)** Correlation of FR^+^CTC level with presence vs. absence of complex glands in the IAC group. **(F)** Correlation of FR^+^CTC level with presence vs. absence of advanced subtypes in the IAC group.

We subsequently performed subgroup analyses of FR^+^CTC level within the IAC group. There was no significant difference between patients with mucinous adenocarcinoma and those with non-mucinous adenocarcinoma [9.93 (7.59-12.77) *vs*. 10.20 (7.18-20.91), *P* = 0.622)] (**Figure 3A**; **Table 1**). In the non-mucinous adenocarcinoma group, no differences were observed between patients whose tumors exhibited different predominant growth patterns [lepidic: 9.74 (7.28-11.90) *vs*. acinar: 10.11 (7.65-12.93) *vs*. papillary: 9.54 (6.97-11.58) *vs*. micropapillary: 11.49 (8.16-13.11) *vs*. solid: 12.42 (8.87-16.37) *vs*. complex gland: 10.22 (7.17-11.77)), *P* = 0.053] (**Figure 3B**; **Table 1**). However, significant differences were observed between groups with and without a micropapillary component [11.21 (8.22-13.61) *vs*. 9.85 (7.43-12.63), *P* = 0.017] (**Figure 3C**; **Table 1**); between groups with and without a solid component [12.16 (8.27-14.90) *vs*. 9.87 (7.50-12.49), *P* = 0.022] (**Figure 3D**; **Table 1**); between groups with and without a complex gland component [10.74 (7.72-13.57) *vs*. 9.87 (7.51-12.69), *P* = 0.405] (**Figure 3E**; **Table 1**); and between the group with a micropapillary, solid, or complex gland component and the group with none of these components [10.48 (7.83-13.67) *vs*. 9.76 (7.42-12.42), *P* = 0.032] (**Figure 3F**; **Table 1**).

**Table 1:** Baseline characteristics of 625 patients with invasive adenocarcinoma.

Characteristics	*N*	FR^+^CTCs (FU/3 ml; median, IQR)	*P*-value
Tumor size (cm)			0.524
0 < T1a ≤ 1	91	10.04 (7.82-12.89)	
1 < T1b ≤ 2	356	10.00 (7.71-12.92)	
2 < T1c ≤ 3	178	9.72 (7.23-12.71)	
Invasive adenocarcinoma			0.622
Mucinous	23	10.20 (7.18-20.91)	
Non-mucinous	602	9.93 (7.59-12.77)	
Predominant histologic subtype of non-mucinous invasive adenocarcinoma			0.053
Lepidic	100	9.74 (7.28-11.90)	
Acinar	354	10.11 (7.65-12.93)	
Papillary	81	9.54 (6.97-11.58)	
Micropapillary	14	11.49 (8.16-13.11)	
Solid	29	12.42 (8.87-16.37)	
Complex glands	24	10.22 (7.17-11.77)	
Presence/absence of histologic pattern			
Lepidic			0.160
Yes	395	9.87 (7.5-12.45)	
No	230	10.35 (7.73-13.57)	
Acinar			0.673
Yes	533	9.93 (7.59-12.69)	
No	92	9.94 (7.54-13.05)	
Papillary			0.078
Yes	271	9.73 (7.31-12.49)	
No	354	10.20 (7.8-13.24)	
Micropapillary			0.017
Yes	103	11.21 (8.22-13.61)	
No	522	9.85 (7.43-12.63)	
Solid			0.022
Yes	60	12.16 (8.27-14.90)	
No	565	9.87 (7.50-12.49)	
Complex glands			0.405
Yes	90	10.74 (7.72-13.57)	
No	535	9.87 (7.51-12.69)	
Advanced subtypes (micropapillary, solid, or complex glands)			0.032
Yes	179	10.48 (7.83-13.67)	
No	446	9.76 (7.42-12.42)	
Degree of differentiation			0.033
Well-differentiated	79	9.75 (6.90-12.01)	
Moderately differentiated	401	9.81 (7.49-12.49)	
Poorly differentiated	145	11.17 (8.01-14.21)	
VPI			0.003
Yes	52	11.53 (8.93-16.56)	
No	573	9.87 (7.43-12.54)	
Lymph node metastasis			0.035
Yes	35	11.21 (9.72-13.53)	
No	518	9.86 (7.49-12.69)	
STAS			0.224
Yes	58	11.21 (8.22-12.84)	
No	567	9.89 (7.49-12.89)	

VPI, visceral pleural invasion; STAS, spread through air spaces.

### 3.4 Correlation of FR^+^CTC level with degree of differentiation, VPI, lymph node metastasis, and spread through air spaces in IAC patients

**Figure 4:**
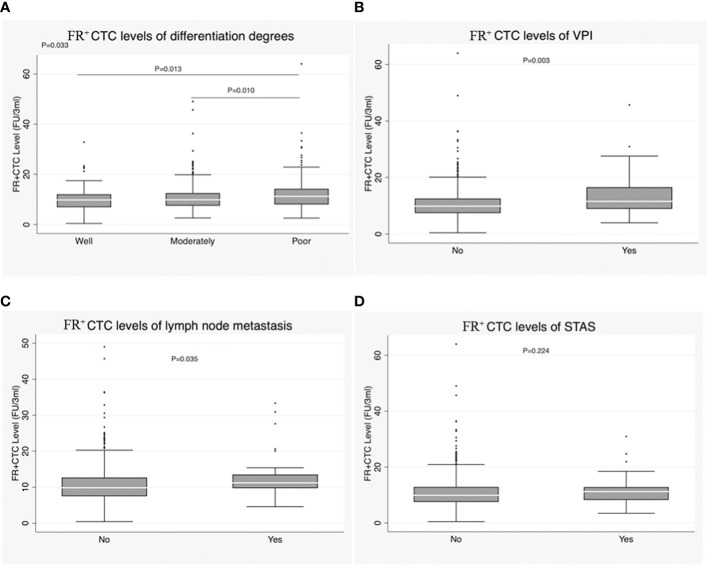
**(A)** Correlation of FR^+^CTC level with degree of differentiation in the IAC group. **(B)** Correlation of FR^+^CTC level with visceral pleural invasion (VPI) in the IAC group. **(C)** Correlation of FR^+^CTC level with lymph node metastasis in the IAC group. **(D)** Correlation of FR^+^CTC level with STAS in the IAC group.

FR^+^CTC level was correlated with degree of differentiation in IAC (*P* = 0.033). Specifically, the median FR^+^CTC level was higher in patients with poorly differentiated tumors than in those with moderately and well-differentiated tumors [11.17 (8.01-14.21) *vs*. 9.81 (7.49-12.49), *P* = 0.010; 11.17 (8.01-14.21) *vs*. 9.75 (6.90-12.01), *P* = 0.013] (**Figure 4A**; **Table 1**). FR^+^CTC level was also correlated with VPI of lung carcinoma [11.53 (8.93-16.56) *vs*. 9.87 (7.43-12.54), *P* = 0.003] (**Figure 4B**; **Table 1**). Additionally, patients with lymph node metastasis had higher FR^+^CTC levels than those without [11.21 (9.72-13.53) *vs*. 9.86 (7.49-12.69), *P* = 0.035] (**Figure 4C**; **Table 1**). However, FR^+^CTC level was not correlated with spread through air spaces (STAS) [11.21 (8.22-12.84) *vs*. 9.89 (7.49-12.89), *P* = 0.224] (**Figure 4D**; **Table 1**).

### 3.5 Correlation of FR^+^CTC level with genetic mutations

We explored the association between FR^+^CTC level and commonly reported driver gene mutations in lung cancer. The results suggested that there was no correlation between FR^+^CTC level and mutations in *EGFR* [9.87 (7.51-12.54) *vs*. 9.82 (7.87-12.69), *P* = 0.775], *ALK* [9.61 (7.16-14.85) *vs*. 10.00 (7.75-12.71), *P* = 0.963], *KRAS* [10.21 (7.86-14.01) *vs*. 10.21 (7.86-14.01), *P* = 0.598], *BRAF* [9.01 (5.67-22.74) *vs*. 9.87 (7.44-13.08), *P* = 0.867], or *ROS1* [10.96 (9.97-11.51) *vs*. 9.87 (7.49-13.05), *P* = 0.494] (**Supplementary Table 2**).

### 3.6 Results of the ROC analysis

**Figure 5:**
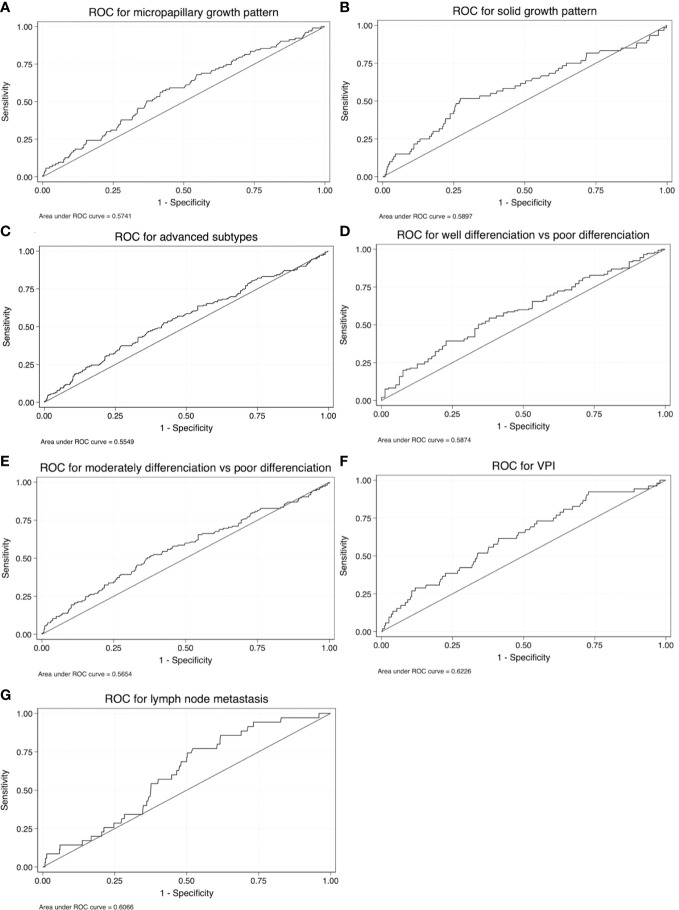
Receiver operating characteristic (ROC) curves for FR^+^CTC level as a test for **(A)** micropapillary components, **(B)** solid components, **(C)** advanced subtypes, **(D)** well-differentiated *vs*. poorly differentiated tumors, **(E)** moderately differentiated *vs*. poorly differentiated tumors, **(F)** VPI, and **(G)** lymph node metastasis.

To further explore the potential clinical value of FR^+^CTC level in distinguishing growth patterns in cases of non-mucinous IAC, an ROC analysis was conducted with two patient groups: all patients with micropapillary component >5% were defined as the case cohort, and patients with micropapillary component ≤5% were designated as a control cohort. The Youden index was used to determine the optimal cutoff value. The ROC analysis yielded an area under the curve (AUC) of 0.574, with a 95% confidence interval (CI) of 0.514-0.634, at the optimal cutoff value of an FR^+^CTC level of 10.40 FU/3 ml. The sensitivity of this test among lung cancer patients was 57.28%, with a specificity of 57.47%, in predicting the presence of micropapillary component >5%, with *P* = 0.009 (**Figure 5A**; **Table 2**).

**Table 2:** Optimal cutoff, sensitivity, specificity, and AUC of ROC curves.

Characteristics	Cutoff	Sensitivity	Specificity	AUC	Lower CI	Upper CI	*P*-value
Micropapillary	10.40	57.28%	57.47%	0.574	0.514	0.634	0.009
Solid	12.13	51.67%	72.74%	0.590	0.506	0.673	0.011
Advanced subtypes	10.40	52.51%	58.07%	0.555	0.504	0.606	0.016
Well differentiated *vs*. poorly differentiated	12.13	39.31%	77.22%	0.587	0.510	0.664	0.015
Moderately differentiated *vs*. poorly differentiated	10.93	51.72%	62.09%	0.565	0.508	0.622	0.010
VPI	10.69	61.54%	58.81%	0.623	0.543	0.702	0.002
Lymph node metastasis	9.72	77.14%	47.88%	0.606	0.523	0.690	0.018

AUC, area under the curve; ROC, receiver operating characteristic; VPI, visceral pleural invasion.

We further explored the use of FR^+^CTC level as a predictor of the presence of solid components, advanced subtypes, degree of differentiation, presence of lymph node metastasis, and VPI. The sensitivity and specificity of FR^+^CTC level for preoperative prediction of the presence of solid components were 51.67% and 72.74%, respectively, and the AUC in this test was 0.590 (95% CI, 0.506-0.673; *P* = 0.011) (**Figure 5B**; **Table 2**). The sensitivity and specificity of FR^+^CTC level for prediction of advanced subtypes were 52.51% and 58.07%, respectively, and the AUC in this test was 0.555 (95% CI, 0.504-0.606; *P* = 0.016) (**Figure 5C**; **Table 2**). The sensitivity and specificity of FR^+^CTC level for distinguishing between well-differentiated and poorly differentiated cases were 39.31% and 77.22%, and the AUC in this test was 0.587 (95% CI, 0.510-0.664; *P* = 0.015) (**Figure 5D**; **Table 2**). Additionally, the sensitivity and specificity of FR^+^CTC level for distinguishing between moderately differentiated and poorly differentiated cases were 51.72% and 62.09%, respectively, and the AUC in this test was 0.565 (95% CI, 0.508-0.622; *P* = 0.010) (**Figure 5E**; **Table 2**). The sensitivity and specificity of FR^+^CTC level for prediction of VPI were 61.54% and 58.81%, respectively, and the AUC in this test was 0.623 (95% CI, 0.543-0.702; *P* = 0.002) (**Figure 5F**; **Table 2**). Finally, the sensitivity and specificity of FR^+^CTC level for prediction of lymph node metastasis were 77.14% and 47.88%, respectively, and the AUC in this test was 0.606 (95% CI, 0.523-0.690; *P* = 0.018) (**Figure 5G**; **Table 2**).

## 4 Discussion

Achieving consistency between intraoperative FS and postoperative final pathology in patients with early-stage lung adenocarcinomas remains a challenging problem. Marchevsky et al. report that intraoperative FS has lower diagnostic accuracy for AIS and MIA than for IAC (51% *vs*. 97%) (25). He et al. have also found that intraoperative FS pathology offers poor differentiation between AAH and AIS and between AIS and MIA. The possible reasons include the deformation of tissues and cells caused by freezing during the operation and the limitations of FS sampling leading to an incomplete representation of the entire lesion (26). In our study, consistency between intraoperative FS and postoperative pathology was only 62.3%, lower than observed in a previous study (64%) (9). The FR^+^CTC test was approved by the NMPA to assist in diagnosing pulmonary lesions and has showed high sensitivity and specificity. In our study, FR^+^CTC level could not be used to distinguish between AIS, MIA, and IAC, which is in line with the results of the study by Ding et al. However, in their study, FR^+^CTCs could be used to distinguish between AIS and MIA (22); this inconsistency of findings may be related to the fact that the participants included in our study were all in the early stage and the sample size was small. Neither FR^+^CTC level nor intraoperative FS can accurately distinguish the pathological subtypes of lung adenocarcinoma. However, larger samples in prospective clinical studies are needed to provide validation in the future.

Some studies have shown that pathological subtype, degree of differentiation, and clinical stage are closely correlated with prognosis in lung cancer (27, 28). However, few studies have explored the ability of FR^+^CTC level to distinguish growth pattern subtypes, as this one has done, and we propose that this measure can be used a reference in determining the extent of surgical resection required in patients with early invasive adenocarcinoma. Adenocarcinoma patients with tumors exhibiting different growth patterns would ideally receive different forms of treatment, and they have different prognoses. In a study by Tsuta et al., 904 cases of surgically resected adenocarcinomas were investigated; it was found that lepidic, acinar, papillary, micropapillary, and solid predominant adenocarcinomas were associated with different prognoses, and their 5-year OS rates were 93%, 67%, 74%, 62%, and 58%, respectively (29). A recent study consisting of 697 patients with pN0M0/papillar/papillar–acinar predominant lung adenocarcinomas with diameter ≤3 cm who underwent curative resection found that the presence of micropapillary and solid patterns as minor components had a negative impact on prognosis. For MP/S^+^ Lep^−^, MP/S^+^ Lep^+^, MP/S^−^ Lep^−^, and MP/S^−^ Lep^+^ types, the 5-year RFS rates were 81.9%, 94.0%, 94.4%, and 98.7%, respectively (*P* < 0.001), and the 5-year OS rates were 87.7%, 96.6%, 94.4%, and 98.4%, respectively (*P* < 0.001). Moreover, a multivariate analysis suggested that the MP/S^+^ Lep^−^ subtype was an independent poor prognostic factor for both RFS and OS (30). The sensitivity and specificity of intraoperative FS for diagnosis of solid primary adenocarcinoma were 79% and 94%, respectively. For the diagnosis of micropapillary primary lung adenocarcinoma, specificity reached 99%, but sensitivity was only 21%. When only the presence or absence of micropapillary subtypes was considered, the diagnostic sensitivity of FS was still only 37% (9).

In our study, we found that there was no difference in level of FR^+^CTCs between the acinar, lepidic, papillary, micropapillary, and solid subtypes in the non-mucinous IAC group. Levels were higher in the acinar subtype compared to the lepidic subtype; in the solid subtype compared to the acinar and papillary subtypes; and in the papillary subtype compared to the lepidic subtype. Furthermore, when only the presence or absence of micropapillary or solid subtypes was considered, there was a notable difference in FR^+^CTC level between the groups (*P* = 0.017 and *P* = 0.022, respectively). The presence of micropapillary or solid patterns was found to significantly increase the risk of nodal upstaging in the multivariable analysis (*P* < 0.001 and *P* = 0.001, respectively), and these patterns were independently associated with poor RFS (*P* = 0.041 and *P* < 0.001) among patients with cT1N0M0 lung adenocarcinoma (14). Micropapillary or solid components have been proven to be poor prognostic factors. Therefore, these patients should be cautious when considering sublobectomy. According to the guidelines, the standard surgery for IAC is still lobectomy; however, in our study, the proportions of patients who underwent lobectomy in high-risk groups with micropapillary and solid components were only 64.1% and 68.3%, respectively (**Supplementary Table 3**). Therefore, it is necessary to identify a biomarker that can aid preoperative pathological judgment and improve the accuracy of selection of surgical method. According to the results of our study, FR^+^CTCs may be a promising auxiliary biomarker for determination of whether solid and micropapillary components are present. However, the diagnostic efficacy is not very good, and the sensitivity and specificity of FR^+^CTC level as a test for the presence of micropapillary components and solid components are relatively low. The AUCs are well below 0.7 (0.574 and 0.590). This means that FR^+^CTC level can only function as a reference in distinguishing whether micropapillary or solid components are present, and does not have a strong ability to make this distinction. Prospective clinical studies with larger samples are needed to provide validation of this finding. The proportion of tumors of the complex gland subtype is small among patients with early-stage adenocarcinoma; thus, there is insufficient evidence to draw a conclusion on its role in prognosis after sublobar resection at present.

FR^+^CTC level was correlated with lymph node metastasis in IAC, with the level being higher in patients with lymph node metastasis than in patients without metastasis (*P* = 0.035), which was consistent with a previous study (27). The sensitivity and specificity of FR^+^CTC level for diagnosis of lymph node metastasis were 77.14% and 47.88%, respectively, and the AUC was 0.606. Thus, at a cutoff value of 9.72 FU/3 ml, this test cannot reliably distinguish whether lymph node metastasis has occurred. Evidence has shown that invasion may occur very early in the tumor process, and CTCs are released into circulation in the early phase of cancer. Lymph node infiltration is related to poor prognosis (28). Many researchers have emphasized the importance of detection of CTCs (31–33). Our results also showed that FR^+^CTC level was correlated with degree of differentiation in IAC. Until now, few studies have focused on the prognostic value of biomarkers in lung tumors with different degrees of differentiation, so this finding is of limited significance because a more accurate method of discrimination has already been developed.

VPI is a high-risk factor that affects prognosis in lung adenocarcinoma. Nevertheless, identification of VPI is still reliant on elastic fiber staining, which is time-consuming and difficult to perform in intraoperative FS. The accuracy, sensitivity, and specificity of intraoperative FS for diagnosis of VPI have been found to be 75%, 47.4%, and 97.3%, respectively (34). Furthermore, our results indicated that FR^+^CTC level was correlated with VPI (*P* = 0.003). However, when 10.25 FU/3 ml was used as the cutoff value, the sensitivity and specificity of FR^+^CTC level for the diagnosis of VPI were 61.54% and 58.81%, respectively. The associated AUC was 0.623, which means that FR^+^CTC is almost capable of distinguishing whether VPI has occurred with sufficient accuracy.

Two studies have shown that the sensitivity of STAS detection in intraoperative FS is only 44%-54%. At the same time, specificity is as high as 80%-91%, and multifactorial logistic regression analysis has found that artifacts are the only relevant factor in the misdiagnosis of STAS by FS (35, 36). However, in the present study, there was no significant difference in FR^+^CTC level between cases with and without STAS (*P* = 0.224). The possible reasons are a relatively small sample size and the inclusion of patients from a population at a specific stage. Ways to improve the sensitivity of STAS detection preoperatively or intraoperatively are worthy of future research.

In our study, we found that the proportions of patients with advanced subtypes (micropapillary or solid or complex glands), lymph node metastasis, poor differentiation, and VPI who underwent lobectomy were 64.8%, 82.9%, 63.5%, and 75.0%, respectively (**Supplementary Table 3**); the proportion did not reach 100% in any of these subgroups. In the case of stage I IAC patients who underwent sublobectomy because high-risk pathological factors (advanced subtypes, VIP, STAS, lymph node metastasis, and so on) could not be identified intraoperatively, postoperative adjuvant therapy or supplementary lobectomy was suggested if the patients agreed (16, 37). FR^+^CTCs can provide helpful information in distinguishing aggressive growth patterns of IAC, lymph node metastasis, and VPI, which could help to identify the optimal surgical strategy and avoid infliction of secondary damage on patients. In the meantime, FR^+^CTC level can be used as a biomarker and possible prognostic factor in early-stage lung adenocarcinoma.

This study had some limitations. The study was preliminary and retrospective in nature, and patient outcomes were not collected and analyzed. In subsequent studies, we will collect long-term prognostic data and analyze the value of FR^+^CTC level in predicting outcomes in early-stage patients. Furthermore, prospective randomized studies are required to truly discern the value of FR^+^CTC level in the preoperative differentiation of histological subtypes and as a tool for use in the selection of surgical method.

## 5 Conclusion

To conclude, FR^+^CTC level may be used as an additional biomarker in identifying patients with micropapillary components, solid components, advanced subtypes, poorly differentiated tumors, VPI, and lymph node metastasis. Lobectomy should be selected as the preferred surgical option for these high-risk patients.

## Data availability statement

The raw data supporting the conclusions of this article will be made available by the authors, without undue reservation.

## Ethics statement

The studies involving human participants were reviewed and approved by the Shanghai Chest Hospital. Written informed consent for participation was not required for this study in accordance with the national legislation and the institutional requirements.

## Author contributions

Conception and design: CZ and WL. Administrative support: AW and WL. Provision of study materials or patients: CZ and WL. Collection and assembly of data: CZ and RZ. Data analysis and interpretation: CZ and RYZ. Manuscript writing and final approval of the manuscript: all authors.
