# Supplementary material for: Preoperative levels of folate receptor-positive circulating tumor cells in different subtypes of early-stage lung adenocarcinoma: Predictive value for determining extent of surgical resection
Source: Front Oncol. 2023 Apr 17;13:1119807. doi: 10.3389/fonc.2023.1119807 (PMC10150082; doi:10.3389/fonc.2023.1119807)
Supplement: Supplementary file 1 [file Table_1.docx]

**Supplementary table 1 Characteristics of patients**

| **Characteristics** | **Final Pathologic Diagnosis** | | | | **P Value** |
| --- | --- | --- | --- | --- | --- |
|  | **Total** | **AIS** | **MIA** | **IAC** |  |
| **N (%)** | 1210 (100.00) | 301 (24.88) | 284 (23.47) | 625 (51.65) |  |
| **Gender (n, %)** |  |  |  |  | <0.001 |
| Male | 437 (36.1) | 80 (26.6) | 97 (34.2) | 260 (41.6) |  |
| Female | 773 (63.9) | 221 (73.4) | 187 (65.8) | 365 (58.4) |  |
| **Age (n, %)** |  |  |  |  | <0.001 |
| <60 years old | 691 (57.1) | 241 (80.1) | 193 (68.0) | 257 (41.1) |  |
| >=60 years old | 519 (42.9) | 60 (19.9) | 91 (32.0) | 368 (58.9) |  |
| **Tumor size (cm, n, %)** |  |  |  |  | <0.001 |
| 0< T1a≤ 1 | 571 (47.2) | 273 (90.7) | 207 (72.9) | 91 (14.6) |  |
| 1< T1b≤ 2 | 459 (37.9) | 27 ( 9.0) | 76 (26.8) | 356 (57.0) |  |
| 2 < T1c≤ 3 | 180 (14.9) | 1 ( 0.3) | 1 ( 0.4) | 178 (28.5) |  |
| **Surgery** (n, %) |  |  |  |  | <0.001 |
| Wedge resection | 284 (23.5) | 137 (45.5) | 85 (29.9) | 62 ( 9.9) |  |
| Segmentectomy | 552 (45.6) | 147 (48.8) | 178 (62.7) | 227 (36.3) |  |
| Lobectomy | 374 (30.9) | 17 ( 5.6) | 21 ( 7.4) | 336 (53.8) |  |
| **Lymph node resection** (n, %) |  |  |  |  | <0.001 |
| No | 321 (26.5) | 159 (52.8) | 90 (31.7) | 72 (11.5) |  |
| Yes | 889 (73.5) | 142 (47.2) | 194 (68.3) | 553 (88.5) |  |
| **Tumor biomarker (median, IQR)** |  |  |  |  |  |
| CEA | 2.33 (1.62-3.33) | 1.98 (1.39-2.95) | 2.00 (1.41-2.90) | 2.65 (1.84-3.60) | <0.001 |
| SCC | 0.64 (0.50-0.84) | 0.62 (0.49-0.78) | 0.66 (0.53-0.86) | 0.64 (0.50-0.87) | 0.032 |
| CYFRA21-1 | 2.16 (1.63-2.77) | 1.94 (1.47-2.54) | 2.12 (1.60-2.74) | 2.27 (1.78-2.91) | <0.001 |
| **FR^+^CTC**  **(FU/3mL, median, IQR)** | 9.93 (7.48-13.0) | 9.92 (7.45-12.7) | 9.86 (7.36-13.4) | 9.93 (7.59-12.9) | 0.813 |

AIS: adenocarcinoma in situ, MIA: minimally invasive adenocarcinoma, IAC: invasive adenocarcinoma, CEA: carcinoembryonic antigen, SCC: squamous cell carcinoma, CYFRA21–1: cytokeratin 19 fragment.

**Supplementary table 2 Correlation of FR^+^CTCs level with genetic mutations.**

| **Characteristics** | **N** | **FR^+^CTC(FU/3ml, median, IQR)** | **P value** |
| --- | --- | --- | --- |
| **Gene mutation status** |  |  |  |
| **EGFR** |  |  | 0.775 |
| Yes | 391 | 9.87 (7.51-12.54) |  |
| No | 75 | 9.82 (7.87-12.69) |  |
| **ALK** |  |  |  |
| Yes | 24 | 9.61 (7.16-14.85) | 0.963 |
| No | 471 | 10.00 (7.75-12.71) |  |
| **KRAS** |  |  | 0.598 |
| Yes | 32 | 10.21 (7.86- 14.01) |  |
| No | 281 | 9.93 (7.49-13.08) |  |
| **BRAF** |  |  | 0.867 |
| Yes | 4 | 9.01 (5.67-22.74) |  |
| No | 287 | 9.87 (7.44-13.08) |  |
| **ROS1** |  |  | 0.494 |
| Yes | 6 | 10.96 (9.97-11.51) |  |
| No | 293 | 9.87 (7.49-13.05) |  |

**Supplementary table 3 The percentage of lobectomy in patients with potential risk factors.**

| **Characteristics** | **Total (n)** | **Sub-lobectomy, n (%)** | **Lobectomy, n (%)** |
| --- | --- | --- | --- |
| **Micropapillary** | 103 | 37 (35.9%) | 66 (64.1%) |
| **Solid** | 60 | 19 (31.7%) | 41 (68.3%) |
| **Advanced subtypes** | 179 | 63 (35.2%) | 116 (64.8%) |
| **Poor differentiated** | 145 | 53 (36.5%) | 92 (63.5%) |
| **VPI** | 52 | 13 (25.0%) | 39 (75.0%) |
| **Lymph node metastasis** | 35 | 6 (17.1%) | 29 (82.9%) |

VPI: Visceral pleural invasion
