# Supplementary figures and images for: Preoperative levels of folate receptor-positive circulating tumor cells in different subtypes of early-stage lung adenocarcinoma: Predictive value for determining extent of surgical resection
Source: Front Oncol. 2023 Apr 17;13:1119807. doi: 10.3389/fonc.2023.1119807 (PMC10150082; doi:10.3389/fonc.2023.1119807)

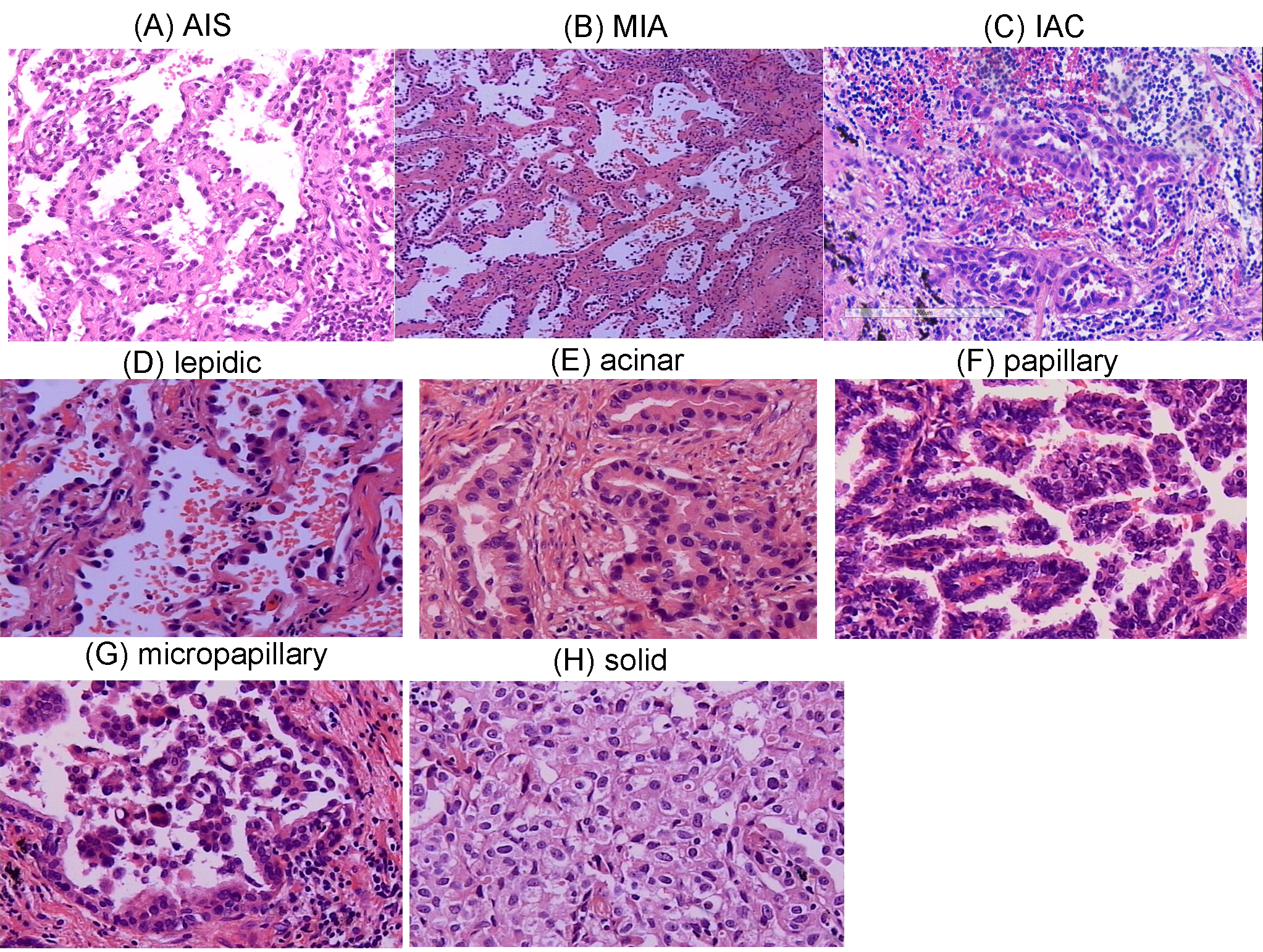

Supplement: Supplementary file 2 [file Image_1.tif]
